# A Phase 2 Study of Sotigalimab, a CD40 Agonist Antibody, plus Concurrent Chemoradiation as Neoadjuvant Therapy for Esophageal and Gastroesophageal Junction Cancers

**DOI:** 10.1158/2767-9764.CRC-24-0513

**Published:** 2025-02-21

**Authors:** Andrew H. Ko, Joseph Chao, Marcus S. Noel, Veena Shankaran, Davendra Sohal, Mary Crow, Paul E. Oberstein, Aaron J. Scott, Autumn J. McRee, Caio Max Sao Pedro Rocha Lima, Lawrence Fong, Bridget P. Keenan, Maira Soto, Erin L. Filbert, Frank J. Hsu, Xiaodong Yang

**Affiliations:** 1 Division of Hematology and Oncology, University of California San Francisco, San Francisco, California.; 2 City of Hope Comprehensive Cancer Center, Duarte, California.; 3 Georgetown University Hospital, Washington, District of Columbia.; 4 University of Washington School of Medicine, Seattle, Washington.; 5 University of Cincinnati, Cincinnati, Ohio.; 6 Renovatio Clinical, Houston, Texas.; 7 Laura and Isaac Perlmutter Cancer Center, NYU Langone Health, New York, New York.; 8 University of Arizona Cancer Center, Tucson, Arizona.; 9 University of North Carolina, Chapel Hill, North Carolina.; 10 Wake Forest University Baptist Medical Center, Winston-Salem, North Carolina.; 11 Pyxis Oncology, Boston, Massachusetts.

## Abstract

**Purpose:**

Neoadjuvant chemoradiation (NCRT) followed by surgical resection represents a standard approach for patients with locally advanced esophageal/gastroesophageal junction (GEJ) cancers. Sotigalimab is a high-affinity CD40 agonist antibody capable of inducing and expanding antitumor immune responses by activating dendritic cells, T and B lymphocytes, NK cells, and M1 macrophages. This study examined the safety and efficacy of combining sotigalimab with NCRT in patients with esophageal or GEJ cancers.

**Patients and Methods:**

Patients with resectable (T1-3 Nx) adenocarcinoma or squamous cell carcinoma of the esophagus or GEJ were eligible. T1N0 and cervical tumors were excluded. Study treatment: weekly carboplatin/paclitaxel with concurrent radiation 5,040 cGy plus 3 to 4 doses of sotigalimab prior to Ivor Lewis esophagectomy. Primary efficacy endpoint was the pathologic complete response (path CR) rate.

**Results:**

Thirty-three patients were enrolled (adenocarcinoma 76%, squamous cell carcinoma 24%; and clinical stage III 67%). Ninety percent of patients received all planned doses of sotigalimab. The most common adverse events attributed to sotigalimab were nausea, fever/chills, fatigue, and cytokine release syndrome; most of these were grade 1 to 2. Grade ≥3 cytokine release syndrome was observed in 3 patients (9%). Twenty-five of the 29 efficacy-evaluable patients underwent an R0 resection (87.9%), with an overall path CR rate of 37.9% (11/29). Post-tumor samples demonstrated increased infiltration and activation of dendritic cells, monocytes, and cytotoxic T cells compared with baseline.

**Conclusions:**

Sotigalimab combined with NCRT for esophageal or GEJ cancers was generally well tolerated and achieved path CR rates that compare favorably with historical data and are promising for this treatment strategy. Clinical trial information: NCT03165994.

**Significance:**

The current study represents the first report to evaluate a CD40 agonist antibody in combination with concurrent chemoradiation in the neoadjuvant setting for patients with esophageal/GEJ cancers. This novel strategy was both safe and feasible, producing encouraging path CR rates that compare favorably with historical data. Our findings support the further evaluation of how immune-based therapies may be incorporated into perioperative treatment paradigms for upper gastrointestinal malignancies.

## Introduction

There are an estimated 22,370 incident cases of esophageal cancer in the United States annually (1), a figure that may in fact underestimate the true number of cases depending on how gastroesophageal junction (GEJ) adenocarcinomas are classified. Trends for histologic subtypes have been shifting, with the incidence of adenocarcinomas steadily climbing over the past several decades in Western countries, and a concomitant decline in squamous cell carcinomas (which still comprise the majority of cases globally; refs. 2, 3).

Multimodal therapy is often employed to optimize clinical outcomes for patients with potentially resectable (localized or locally advanced) esophageal/GEJ cancers, which can include either perioperative chemotherapy, preoperative chemoradiation, or some combination of these different strategies. A common approach consists of daily radiation over a 5- to 6-week course, administered preoperatively, concurrently with low-dose weekly carboplatin and paclitaxel as informed by the CROSS trial, a Dutch phase 3 study that demonstrated a significant disease-free and overall survival benefit with trimodality therapy compared with surgery alone in patients with T1N1 and T2-3 N0-1 tumors of both squamous cell and adenocarcinoma histology (4).

Immunotherapy, primarily in the form of immune checkpoint inhibitors, now has an established role in the treatment of gastroesophageal cancer, with anti-PD1 monoclonal antibodies being approved for use in combination with chemotherapy in the first-line setting for patients with advanced and metastatic disease (5, 6), as well as for earlier stage disease in the postoperative adjuvant setting in patients who did not achieve a pathologic complete response (path CR) to neoadjuvant chemoradiation (7). A number of studies have also explored or are exploring the potential benefits of immunotherapy in the neoadjuvant setting, including several randomized trials evaluating the efficacy of adding an anti-PD(L)1 monoclonal antibody to chemotherapy in this clinical context (8, 9). However, to date this does not represent an approved standard of care. Other immunotherapeutic strategies beyond immune checkpoint inhibition have been less well studied in gastroesophageal cancer, particularly their potential for being incorporated into treatment paradigms for nonmetastatic disease.

CD40 is a member of the tumor necrosis factor receptor superfamily and is expressed by both immune and nonimmune cells, including antigen-presenting cells (APC) such as dendritic cells, B lymphocytes, and macrophages, as well as a variety of epithelial malignancies (10, 11). Binding of this receptor by the CD40 ligand (CD154) promotes the maturation of dendritic cells and induces upregulation of MHC molecules as well as a host of critical T-cell stimulatory cytokines, such as IL-12, resulting in enhanced antigen presentation and activation of CD8^+^ T cells. Sotigalimab (Pyxis Oncology), a humanized CD40-agonistic antibody, binds to CD40 with high affinity (12) and was demonstrated to produce dose-dependent increases in APCs and T-cell activation in a first-in-human study in subjects with advanced solid tumors, consistent with CD40 engagement (13, 14). It has since been evaluated in several tumor types in combination with both chemotherapy and immune checkpoint inhibitors (14–16). We designed a phase 2 trial to evaluate the addition of sotigalimab to standard chemoradiation (per the CROSS trial) in patients with resectable esophageal and GEJ cancer. The correlative findings from this study, describing the immunologic effects of CD40 agonism in a small cohort of subjects who received a single dose of sotigalimab prior to initiation of chemoradiation as part of the safety lead-in phase, have been published previously (17). The present report details the safety and efficacy of this novel combination in the entire study cohort.

## Patients and Methods

### Study design

This was a multicenter, non-randomized, single-arm, open-label trial conducted at 7 centers across the United States in accordance with the Declaration of Helsinki. Institutional review board approval was obtained at each site. All participants provided written informed consent. Primary study objectives were to assess the path CR rate at the time of surgery following study treatment consisting of sotigalimab × 3 to 4 doses in combination with radiation plus weekly carboplatin and paclitaxel over 5 to 6 weeks; and to establish the safety and feasibility of this novel regimen when administered in the neoadjuvant setting. The study included a safety run-in phase for the first 6 patients, in which a number of molecular and immune-based biomarkers and immune cell infiltrates were measured at baseline and following a single dose of sotigalimab prior to initiation of concurrent chemoradiation.

### Patient population

To be eligible for this study, patients were required to be 18 years and older with a histologically confirmed diagnosis of squamous cell carcinoma, adenocarcinoma, or undifferentiated carcinoma of the esophagus or GEJ, clinically staged as T1-3 Nx by endoscopic ultrasound. Subjects with very early stage disease (T1N0), tumors of the cervical esophagus, or tumors invading the tracheobronchial tree or associated with tracheoesophageal fistula were excluded, as were those with cervical, supraclavicular, or other nodal disease not included in the radiation field or not able to be resected at the time of esophagectomy. Patients were required to have an Eastern Cooperative Oncology Group performance status of 0 to 1 with no medical contraindications to undergoing surgical resection and have adequate hematologic, renal, and hepatic parameters defined as follows: absolute neutrophil count ≥1.5 × 10^9^/L, platelet count ≥150 × 10^9^/L, hemoglobin >9 g/dL, serum creatinine ≤1.5 mg/dL, or creatinine clearance ≥30 mL/minutes, aspartate aminotransferase (AST) and alanine aminotransferase (ALT) ≤2.5 × upper limit of normal (ULN), and total bilirubin ≤1.5 × ULN. Other key exclusion criteria included a history of autoimmune disorders (excepting vitiligo or autoimmune thyroid disorders), chronic steroid dependency (prednisone equivalent >10 mg/day), preexisting peripheral sensory neuropathy greater than grade 2, or significant active cardiac disease.

### Study treatment and assessments

All patients received standard chemoradiation consisting of carboplatin (AUC 2) plus paclitaxel (50 mg/m^2^ intravenously) weekly for 5 weeks, concurrent with daily radiation (Monday through Friday) to a total of 5,040 cGy to the planning target volume in 28 fractions. Although this dose of radiation is higher than the 4,140 cGy administered in the CROSS trial, it reflects contemporary clinical practice and represented the standard of care radiation dosing at each of the participating sites.

The starting dose of sotigalimab was 0.3 mg/kg, which was two dose levels below the highest safe dose identified in the completed phase 1 study (1 mg/kg), and was chosen owing to a favorable safety profile and known immune stimulation activity at that level. Prior to each dose of sotigalimab, patients were premedicated with an oral H1 antagonist (e.g., loratadine 10 mg), ibuprofen, acetaminophen, and an optional oral H2 antagonist and then monitored for 4 hours afterward.

**Figure 1:**
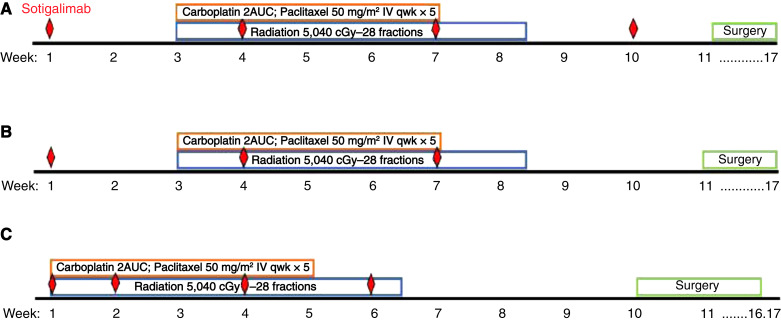
Treatment schema demonstrating changes in timing and dosing intervals of sotigalimab relative to the administration of neoadjuvant chemoradiation and surgery, based on sequential protocol amendments. Number of patients treated in each cohort: cohort A (*n* = 3); cohort B (*n* = 10); and cohort C (*n* = 20).

The protocol underwent sequential amendments for pragmatic and clinical reasons over time, leading to adjustments in the number of doses, timing and intervals of sotigalimab treatment, as highlighted in Fig. 1. In the original study schema (cohort A; *n* = 3), patients received a single “run-in” dose of sotigalimab prior to initiation of chemoradiation, followed by an additional 3 doses of sotigalimab on an every 3-week schedule (4 doses total) during and following completion of chemoradiation. The protocol was subsequently amended (cohort B; *n* = 10) to eliminate the fourth and final dose of sotigalimab owing to surgical concerns about receipt of a pro-inflammatory agent so soon prior to major surgery. With the second amendment (cohort C; *n* = 20), the run-in dose of sotigalimab was omitted to allow patients to start chemoradiation in as expeditious fashion as possible, while also evaluating a dose-intensified schedule of sotigalimab (weeks 1, 2, 4, and 6 concurrent with chemoradiation; 4 doses total). When administered concurrently with chemoradiation, sotigalimab was infused on a separate day (offset by 2–3 days) from carboplatin and paclitaxel to avoid the potential counteracting effects of steroid premedication.

Repeat imaging (CT-PET scan) was performed a minimum of 4 weeks, and Ivor Lewis esophagectomy was performed within 10 weeks, following completion of chemoradiation. Surveillance imaging occurred at months 3 and 6 after surgery.

### Statistical methodology

A total of 30 efficacy-evaluable subjects, defined as patients who met all study eligibility criteria, received at least one dose of sotigalimab, and underwent surgical resection, were to be enrolled. The null hypothesis that the true path CR is 29% (based on data reported from the CROSS trial; ref. 4) was tested against a one-sided alternative hypothesis of 53% using an exact binomial test, with a type 1 error rate of 0.05 and power of 81%. Other efficacy data (secondary endpoints) that were captured include rates of R0 resection, pathologic stage at time of surgery, and radiographic/metabolic response to neoadjuvant treatment on CT-PET. As this patient population does not typically have measurable disease by RECIST, the radiographic/metabolic response was described purely in qualitative terms (i.e., improved/stable/worse). These data were reported both for the entire study cohort and for each histologic subgroup.

All patients who received at least one dose of sotigalimab were included in the safety population. Safety data were collected up to 3 months following surgery (longer if there were any lingering adverse events (AE) directly related to study treatment) and reported in summary fashion based on the frequency of AEs, tabulated by grade and organ system using the NCI-Common Terminology Criteria for Adverse Events v4.03.

### Correlative studies

Tumor biopsies and peripheral blood samples were collected at baseline, following an initial dose of sotigalimab, and following chemoradiation at the time of surgery from six patients during the safety lead-in portion of the study. High-dimensional single-cell techniques were used, including combined single-cell RNA sequencing and proteomics and multiplexed ion beam imaging, to analyze immune responses. Details of the methodology of this immune-based correlative work have been reported previously (17).

### Data availability

The data generated in this study are available upon request from the corresponding author.

## Results

### Patient demographics and disease characteristics

Demographic and baseline characteristics are summarized in Table 1. The median age of the study population was 67 years, and the majority of participants were male, consistent with the demographics associated with this disease type. Two thirds of subjects (67%) had an Eastern Cooperative Oncology Group performance status of 0 at baseline. The representativeness of our study cohort is described in Supplementary Table S1.

**Table 1:** Patient demographics and baseline tumor characteristics

Parameter	Total (*N* = 33)
Sex, *n* (%)	
Female	7 (21.2)
Male	26 (78.8)
Age, years	
Mean (SD)	64.5 (9.17)
Median (Q1, Q3)	67.0 (59.0, 71.0)
Minimum, Maximum	38, 75
Race, *n* (%)	
White or Caucasian	25 (75.8)
Asian	5 (15.2)
Black	1 (3.0)
Eastern Cooperative Oncology Group baseline	
0	22 (66.7)
1	11 (33.3)
Tumor staging assessment histology	
Squamous cell carcinoma	8 (24.2)
Adenocarcinoma	25 (75.8)
Grade	
Missing	0
Grade X	2 (6.1)
Grade 1	1 (3.0)
Grade 2	20 (60.6)
Grade 3	10 (30.3)
Tumor location	
Upper third of the esophagus	2 (6.1)
Middle third of the esophagus	8 (24.2)
Lower third of the esophagus	8 (24.2)
GEJ	15 (45.5)
T stage	
Missing	0
T2	6 (18.2)
T3	27 (81.8)
N stage	
N0	8 (24.2)
N1	17 (51.5)
N2	7 (21.2)
N3	1 (3.0)
Stage group (cTNM/TNM)	
Missing	0
II	2 (6.1)
IIB	1 (3.0)
III	22 (66.7)
IVA	8 (24.2)

From a standpoint of tumor histology, 25 subjects (76%) had adenocarcinoma and 8 (24%) had squamous cell carcinoma. The majority of patients had cancers located in the distal esophagus or GEJ (70%). Most tumors were clinically staged as T3 (81.8%) and/or had involved lymph nodes (75.7%), reflecting the higher stage of disease most patients presented with in this clinical trial.

### Safety

All 33 subjects who received the study drug were included in the safety population. The most common non-hematologic treatment-emergent AEs (TEAE) of any grade were fatigue, nausea, constipation, diarrhea, chills, and transaminase elevation (Table 2). Grade 3 or higher TEAEs were observed in 26 subjects (78.8%), with neutropenia and leukopenia representing the most frequently observed grade 3 to 4 toxicities, both occurring in 24.2% of patients. By contrast, grade 3 to 4 non-hematologic toxicities occurred relatively infrequently, with no individual grade 3+ AE occurring in more than 10% of subjects (Table 2). Furthermore, no TEAEs led to withdrawal from the study, and no deaths were attributable directly to study treatment. The one grade 5 TEAE observed on this clinical trial was an episode of aspiration pneumonia that occurred in the postoperative setting and deemed unrelated to study treatment.

**Table 2:** Most common TEAEs (occurring in ≥10% of subjects; *n* = 33)

	Any grade	Grade 3 or higher
	*n* (%)	*n* (%)
At least one event	33 (100)	19 (57.6)
Hematologic		
Thrombocytopenia	15 (45.5)	3 (9.1)
Leukopenia	14 (42.4)	8 (24.2)
Neutropenia	14 (42.4)	8 (24.2)
Anemia	9 (27.3.)	1 (3.0)
Lymphopenia	5 (15.2)	4 (12.1)
Gastrointestinal disorders		
Nausea	23 (69.7)	3 (9.1)
Constipation	16 (48.5)	0 (0)
Diarrhea	15 (45.5)	0 (0)
Vomiting	13 (39.4)	3 (9.1)
Dysphagia	11 (33.3)	3 (9.1)
Esophagitis	11 (33.3)	3 (9.1)
Esophageal pain	7 (21.2)	0 (0)
Odynophagia	6 (18.2)	0 (0)
Dyspepsia	4 (12.1)	0 (0)
General disorders and administration site conditions		
Fatigue	24 (72.7)	1 (3)
Chills	16 (48.5)	0 (0)
Pyrexia	8 (24.2)	0 (0)
Hepatobiliary disorders		
Hyperbilirubinemia	4 (12.1)	0 (0)
Immune system disorders		
CRS	11 (33.3)	3 (9.1)
Injury, poisoning, and procedural complications		
Infusion-related reaction	7 (21.2)	0 (0)
Investigations		
ALT increased	14 (42.4)	2 (6.1)
AST increased	14 (42.4)	2 (6.1)
Alkaline phosphatase increased	6 (18.2)	0 (0)
Weight decreased	4 (12.1)	0 (0)
Metabolism and nutrition disorders		
Hyponatremia	9 (27.3)	2 (6.1)
Decreased appetite	8 (24.2)	1 (3.0)
Dehydration	8 (24.2)	2 (6.1)
Hypokalemia	6 (18.2)	2 (6.1)
Hypomagnesemia	4 (12.1)	0 (0)
Musculoskeletal and connective tissue disorders		
Arthralgia	4 (12.1)	1 (3.0)
Nervous system disorders		
Headache	7 (21.2)	0 (0)
Respiratory, thoracic, and mediastinal disorders		
Cough	5 (15.2)	0 (0)
Dyspnea	5 (15.2)	0 (0)
Dysphonia	4 (12.1)	0 (0)
Epistaxis	4 (12.1)	0 (0)
Pruritus	5 (15.2)	0 (0)
Alopecia	4 (12.1)	0 (0)
Hypotension	7 (21.2)	1 (3.0)

Thirty subjects (90.9%) experienced at least one sotigalimab-related TEAE that generally occurred during or within 72 hours after the infusion of sotigalimab. These AEs were often reported according to their predominant symptoms (i.e., fatigue, chills, or nausea/vomiting) rather than as an infusion-related reaction or cytokine release syndrome (CRS; Table 3). Although the majority of events were grade 1 or 2, three subjects (9.1%) experienced grade 3 CRS; of these, one was treated with tocilizumab, whereas the other two improved with supportive care measures alone including intravenous hydration, acetaminophen, and/or ketorolac. Serious AEs of CRS led to a dose reduction of sotigalimab in 2 patients, whereas one nonserious AE of CRS led to a dose interruption. Three subjects were hospitalized for close monitoring and management of their CRS. All events of CRS fully resolved, typically within 24 to 48 hours.

**Table 3:** Most common AEs (occurring in ≥10% of subjects) considered related to sotigalimab by the investigator (*n* = 33)

AE	Total *n* (%)
Fatigue	16 (48.5)
Chills	15 (45.5)
Nausea	15 (45.5)
CRS	11 (33.3)
AST increased	8 (24.2)
Vomiting	8 (24.2)
Diarrhea	7 (21.2)
Pyrexia	7 (21.2)
Thrombocytopenia	7 (21.2)
ALT increased	6 (18.2)
Decreased appetite	5 (15.2)
Hypotension	5 (15.2)
Infusion-related reaction	5 (15.2)
Pruritus	5 (15.2)
Arthralgia	4 (12.1)
Blood alkaline phosphatase increased	4 (12.1)
Headache	4 (12.1)
Leukopenia	4 (12.1)

An additional 3 subjects had grade 4 AEs that were considered related to sotigalimab: lymphopenia in 2 subjects and Guillain–Barre Syndrome in 1 subject. The participant with grade 4 Guillain–Barre Syndrome, who had received their last dose of sotigalimab (0.3 mg/kg) 60 days prior to the onset of neurologic symptoms, required prolonged hospitalization with ventilatory and vasopressor support and intravenous immunoglobulin but made a gradual neurologic recovery over the course of the next several months.

Patients did not experience a higher postoperative complication rate than expected with this type of cancer operation, with no reported cases of mediastinitis, anastomotic leak, or cardiac events.

### Treatment administration and efficacy

Thirty of 33 patients (90.1%) received their full planned doses of sotigalimab (either 3 or 4 infusions, depending on the cohort). Six (18.2%) participants had at least one TEAE that led to either interruption or, in the case of 3 subjects, omission of 1 or 2 doses of sotigalimab. In terms of neoadjuvant chemoradiation, 31 patients (93.9%) were able to complete their full prescribed course of radiation; one patient missed one daily fraction of treatment and another discontinued radiation after a total of 18 fractions; 96.9% received all 5 weekly doses of carboplatin and paclitaxel; and a single patient missed one dose of carboplatin. The median time from completion of neoadjuvant treatment to surgical resection was 2 months. Four of 33 subjects (12.1%) in the entire study cohort did not go onto surgery, including 3 who declined an operation and 1 who passed away prior to surgery secondary to complications associated with an unrelated mechanical fall.

The primary efficacy measure was the proportion of patients who achieved a path CR. Of the 29 patients who were taken to surgery (representing the efficacy-evaluable population), 11 (37.9%) had a path CR, including 3 of 5 (60%) with squamous cell carcinoma and 8 of 24 (33%) with adenocarcinoma (Table 4). An additional 8 subjects (28.6%) were classified as having a major pathologic response, defined as <10% remaining residual cancer cells present.

**Table 4:** Efficacy results

	Efficacy population	Histologic subtype		Location
		Adenocarcinoma	Squamous cell	GEJ
	*n* = 29	*n* = 24	*n* = 5	*n* = 14
	% (*n*/total)	% (*n*/total)	% (*n*/total)	% (*n*/total)
R0 resection	86.2% (25/29)	83.3% (20/24)	100% (5/5)	78.6% (11/14)
Pathologic response				
Complete response (path CR)	37.9% (11/29)	33.3% (8/24)	60.0% (3/5)	28.6% (4/14)
Major pathologic response^a^	65.5% (19/29)	62.5% (15/24)	80.0% (4/5)	57.1% (8/14)
Progressive disease (before or at surgery)	6.9% (2/29)	8.3% (2/24)	0% (0/5)	14.3% (2/14)

a Major pathologic response defined as <10% viable tumor that includes both pathologic complete and partial responses.

Other secondary endpoints included (i) R0 resection rate, which was achieved in 25 of 29 subjects (86.2%), and (ii) radiographic response to neoadjuvant treatment on protocol-mandated CT-PET performed following completion of chemoradiation and prior to planned surgery. As this patient population typically did not have RECIST-measurable disease, the imaging endpoint was described in purely qualitative terms, with subjects’ radiographic response placed into one of three general categories: improved, stable, or worse. Twenty-two subjects (75.9%) were reported as showing improvement, with an additional 5 (17.2%) demonstrating stable disease.

Rates of relapse-free and overall survival were not captured as clinical endpoints in this study. At the time of database lock, with a median follow-up time of 429 days (range, 327–1,393), 7 subjects (21.2%) had died: 5 (15.2%) because of disease progression/recurrence, 1 related to postoperative complications (aspiration pneumonia), and 1 because of unrelated causes.

## Discussion

In this multicenter phase 2 study, we demonstrate that the addition of sotigalimab to concurrent chemoradiation is both safe and feasible for patients with resectable esophageal and GEJ cancer. In addition to its high binding affinity to CD40 (1.2 × 10^−10^ mol/L), sotigalimab was specifically designed to optimize interaction selectively with Fcγ receptors, including enhanced binding to FcγRIIb, which increases crosslinking of sotigalimab, leading to enhanced agonist activity by FcR-bearing cells, while eliminating binding to Fcγ IIIa and antibody-dependent cellular cytotoxicity effector function of sotigalimab to prevent antibody-dependent cellular cytotoxicity–mediated elimination of CD40-expressing APCs (12, 17). Interaction of sotigalimab with CD40 triggers the cellular proliferation and activation of APCs, including B lymphocytes, dendritic cells, macrophages, and monocytes, leading to the generation of both T cell–dependent as well as humoral immune responses against tumor cells. CD40 agonists have also been shown to increase sensitivity to immune checkpoint blockade (18, 19), with sotigalimab and others in its class demonstrating promise when tested in combination with anti-PD1 antibodies in clinical trials for melanoma and other disease indications (14, 15, 20), including patients who have developed resistance to prior anti-PD1 therapy (17). Finally, CD40 is present on the surface of various epithelial tumor cells (21), including both gastric adenocarcinomas (22) and esophageal squamous cell carcinomas (23), suggesting an additional mechanism of action of CD40 agonists via direct induction of tumor cell apoptosis.

To date, a strategy of administering immunotherapy, in particular anti-PD1 or anti-PDL1 antibodies, concurrently with chemoradiation for esophageal/GEJ cancer has been explored in only a limited number of clinical trials. Multiple lines of preclinical and clinical evidence support synergistic activity between radiotherapy and immunotherapy; specifically, activation of immune cells by immune checkpoint inhibitors may induce more robust local tumor regression and provide improved systemic control both by reprogramming the tumor microenvironment (TME) to increase the radiosensitivity of tumors and by stimulating off-target abscopal effects (24–27). In a phase 2 feasibility trial (PERFECT) reported by Van Den Ende and colleagues (28), patients with resectable esophageal adenocarcinoma received neoadjuvant chemoradiation according to the CROSS regimen combined with atezolizumab during and following chemoradiation. The completion rate of all five cycles of atezolizumab, which represented the primary study endpoint, was achieved in 34 of 40 (85%) of patients, with 33 ultimately undergoing surgery; path CR rate for the cohort was 25%. In a similarly designed phase 1b/2 study by Zhu and colleagues (29) evaluating pembrolizumab × 2 to 3 doses in combination with CROSS-based chemoradiation for patients with GEJ adenocarcinoma, 29 of 31 patients (90%) received all expected doses of immunotherapy and 28 underwent R0 resection, with a path CR rate of 22.6%. Schlosser and colleagues (30), in their RICE trial (*n* = 56), combined durvalumab (×2 cycles) with the CROSS regimen for patients with operable esophagogastric adenocarcinoma; 53 subjects (95%) completed immuno-chemoradiation per protocol and 100% proceeded to surgery, with an observed path CR rate of 23.6%.

Most recently, results from the phase 2/3 EA2174 cooperative group study were reported by Eads and colleagues (31), in which 275 patients with resectable esophageal/GEJ adenocarcinoma were randomized to receive neoadjuvant CROSS either with or without concurrent nivolumab × 2 doses (a second randomization in the study evaluated 6–12 months of nivolumab with or without 6 months of ipilimumab in the postoperative adjuvant setting). The primary neoadjuvant endpoint, path CR rate, showed no statistically significant difference between the nivolumab- and non-nivolumab–containing arms (24.8% vs. 21.0%; *P* = 0.27). Surgical complication rates between the two arms were similar.

To our knowledge, ours is the first study to use an alternative immunotherapeutic approach besides immune checkpoint inhibitors in conjunction with chemoradiation as neoadjuvant therapy for esophageal and GEJ tumors. Although CD40 activation may further act in nonredundant fashion from radiation to augment antitumor immunity and help overcome resistance to immune checkpoint blockade (32), our study intentionally did not include an immune checkpoint inhibitor to avoid evaluating too many disparate, and potentially toxic, components within the experimental regimen in this potentially curative setting. Nevertheless, findings from this non-randomized clinical trial show that the novel combination produced pathologic responses that compare favorably with historical controls of chemoradiation without immunotherapy. Specifically, the original phase 3 trial establishing the CROSS regimen as a neoadjuvant standard of care reported a path CR rate of 29% (squamous cell 49% and adenocarcinoma 23%). This same CROSS regimen was associated with lower path CR rates (10%–14%) when used in more recent randomized phase 3 trials focusing exclusively on adenocarcinoma histologic subtype (29, 30). Acknowledging the small sample size and non-randomized design of the current study that limits definitive interpretation, the path CR rates of 33% and 60% for adenocarcinoma and squamous cell carcinoma, respectively, in our study do suggest a possible additive antitumor effect of sotigalimab in this neoadjuvant context.

The safety profile, and potential additive or synergistic toxicities, of administering a novel immunotherapeutic agent concurrently with chemoradiation warrants special attention, especially in this preoperative (and potentially curative) setting. Predictably, the majority of patients on our study did experience an AE attributable to sotigalimab, including 33% with CRS (which likely underestimates the true incidence, depending on how AEs were reported and classified). However, most of these CRS events were categorized as grade 1 or 2, were readily managed on an outpatient basis, and resolved rapidly. Meanwhile, given the modest sample size of our study, it is difficult to ascertain whether the addition of sotigalimab resulted in a greater frequency or severity of toxicities commonly associated with chemoradiation. For example, AEs (any grade) of fatigue (72.7%) and decreased appetite (24.2%) in our cohort were fairly similar to those reported in the CROSS trial, whereas nausea (69.7%), vomiting (39.4%), and esophagitis (33.3%) were observed somewhat more frequently compared with those in the CROSS trial. Moreover, we did observe in our study a slightly higher incidence of certain grade 3 or higher AEs, including esophagitis and nausea, again acknowledging the difficulties of cross-study comparisons and noting that these each still occurred in fewer than 10% of subjects. Reassuringly, the postoperative in-hospital mortality rate observed in our study (1 of 29 subjects, or 3.4%) was similar to that reported in the CROSS trial in subjects receiving standard chemoradiation (2.4%), without higher incidences of anastomotic leakage, chylothorax, mediastinitis, or postoperative pulmonary or cardiac complications.

To emphasize, although data from the current clinical trial and the other abovementioned trials demonstrate both the feasibility of combining immunotherapy with chemoradiation in the neoadjuvant setting and moderate clinical activity, the modest sample size of our trial and absence of longer-term follow-up data on disease-free and overall survival limit the ability to draw firmer conclusions regarding the robustness of this approach. Moreover, patients on our study were not treated in uniform fashion owing to sequential protocol amendments leading to changes in the dose intensity and administration of sotigalimab over time, raising additional questions regarding optimal dose administration of this treatment strategy. It is worth noting that in a recent phase 3 trial combining immunotherapy with chemotherapy in the perioperative setting for patients with resectable esophagogastric cancer, improvements observed in the path CR rate did not translate into a significant benefit in event-free or overall survival (8), raising the important question of whether using path CR as the primary efficacy endpoint in our trial represents an appropriate surrogate for more clinically meaningful oncologic outcomes. Furthermore, although our inclusion of both squamous cell carcinomas and adenocarcinomas was intended to mirror eligibility for the CROSS trial, one could argue that contemporary trials in this disease setting should focus exclusively on a single histology and that our trial was insufficiently powered to tease out the efficacy in each individual histologic subtype.

The safety run-in cohort from our clinical trial does provide some valuable insights into the on-target immunologic effects of sotigalimab that are worth exploring further in this and/or other disease contexts. Specifically, as reported previously by Soto and colleagues (17), we observed that sotigalimab dramatically remodeled the immune compartment in the periphery and within the TME, increasing expression of molecules related to antigen processing and presentation, priming new T-cell clonotypes with increased density and activation of T cells with enhanced cytotoxic function in the TME, while simultaneously decreasing the frequency of regulatory T cells. Predictive biomarkers at a genetic level, and/or baseline composition of immune cell compartments both within the tumor and in peripheral circulation, should ideally be embedded into future study design to identify the subgroup of patients most likely to benefit from this enhanced strategy.

In conclusion, the current study provides compelling evidence for the efficacy of a CD40 agonistic antibody in combination with neoadjuvant chemoradiation in esophageal cancer, highlighting its potential to improve clinical outcomes via enhanced immune responses. Incorporation of immunotherapeutic agents into perioperative treatment paradigms, whether administered concomitantly with chemotherapy ± radiation or sequenced before or after these modalities, remains an attractive approach and represents an area of active investigation. Looking to the future in both clinical practice and trial design, two recent randomized phase 3 trials indicate that perioperative chemotherapy confers equivalent or superior survival outcomes when compared with neoadjuvant chemoradiation in patients with locally advanced esophageal and GEJ cancer (33, 34), suggesting that, more broadly speaking, the standard approach may be shifting more in the direction of systemic therapy only (minus radiation) for this patient population. Further evaluation of novel immunotherapies, not only added to but perhaps in certain situations also supplanting standard treatment, is warranted in this clinical context.

## Supplementary Material

Supplementary Table 1 Representativeness of Study Participants Table
