# Supplementary material for: A Phase 2 Study of Sotigalimab, a CD40 Agonist Antibody, plus Concurrent Chemoradiation as Neoadjuvant Therapy for Esophageal and Gastroesophageal Junction Cancers
Source: Cancer Res Commun. 2025 Feb 21;5(2):349–57. doi: 10.1158/2767-9764.CRC-24-0513 (PMC11843624; doi:10.1158/2767-9764.CRC-24-0513)
Supplement: Supplementary Table 1 — Representativeness of Study Participants Table [file crc-24-0513_supplementary_table_1_suppst1.docx]

| **Supplementary Table 1.** Representativeness of Study Participants | |
| --- | --- |
| Cancer type(s)/subtype(s)/stage(s)/condition | Esophageal and GE junction cancers (adenocarcinoma and squamous cell carcinoma) |
| Considerations related to: | |
| Sex | Esophageal and GE junction cancers have a male predominance, with an approximately 4:1 male to female ratio based on data from the National Cancer Institute's (NCI's) Surveillance, Epidemiology, and End Results (SEER) program and the Centers for Disease Control and Prevention's National Program of Cancer Registries (NPCR). This ratio is higher for adenocarcinomas (9:1) than squamous cell carcinomas (2-3:1). |
| Age | Esophageal and GE junction cancers are most frequently diagnosed among people aged 65–74, with a median age of 68 years. |
| Race/ethnicity | In the USA from 2017 to 2021, the overall esophageal cancer incidence rate by race and ethnicity was as follows (rates of new cases per 100,000 persons):   - Non-Hispanic whites: 8.3 (male), 1.9 (female) - Non-Hispanic blacks: 5.2 (male), 1.9 (female) - Non-Hispanic Asian/Pacific Islander: 3.6 (male), 1.0 (female) - Non-Hispanic American Indian/Alaskan Native: 9.0 (male), 2.2 (female) - Hispanic: 4.7 (male), 1.0 (female) |
| Geography | In the US, there will be an estimated 22,070 new cases of esophageal cancer diagnosed in 2025 and about 16,250 individuals will die from this disease. Worldwide, more than 600,000 patients were diagnosed with esophageal cancer in 2020, with about 544,000 deaths; the highest incidence rates are seen in Eastern Asia and Southern and Eastern Africa.  In California, where this clinical trial originated and the greatest number of subjects were enrolled, the average annual count (2017-21) was 1,580, with an age-adjusted incidence rate of 3.5 cases per 100,000 (compared to 4.5 nationwide). |
| Other considerations | The incidence of esophageal cancer has declined in the United States since the early 2000s. The histologic distribution has also shifted, with the incidence of adenocarcinoma overtaking that of squamous cell carcinoma in 1995. Overall demographic trends show that the greatest rate of increase of esophageal cancer cases during the past 2 decades has been observed in non-Hispanic White male individuals compared with other racial and ethnic groups, particularly those age 75 and above. |
| Overall representativeness of this study | The age distribution (38 – 75; median 67 years) of our study very closely approximated nationwide statistical data, as did our male:female ratio (4:1).  The generalizability of these study findings is limited by underrepresentation of Black patients (3%). There was an overrepresentation of Asian patients (15%), which partially reflects the catchment area of the highest enrolling site (San Francisco/Bay Area). |
